# In This Issue

**DOI:** 10.1111/cas.70478

**Published:** 2026-07-18

Volume 117, Issue 8, August 2026

## Neutrophil‐Macrophage Interactions Shape Inflammatory Macrophage Remodeling in Gastric Cancer During Chemo‐Immunotherapy

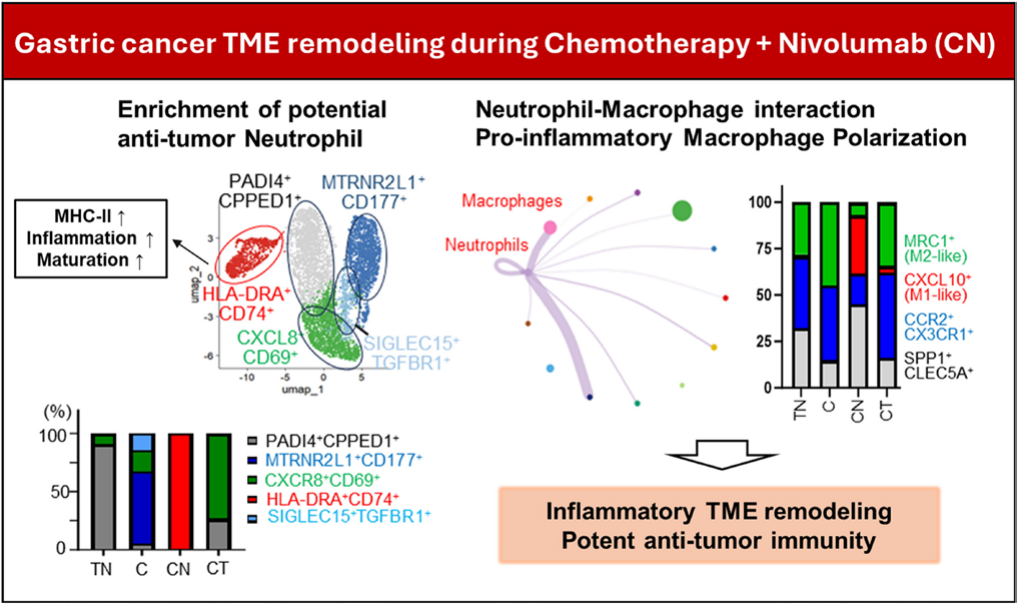

Stomach cancer (also known as gastric cancer) remains one of the world's deadliest cancers, and many patients do not respond equally well to available treatments. While chemotherapy and immunotherapy can improve outcomes for some patients with stomach cancer, the immune mechanisms that contribute to these responses are not fully understood. In particular, scientists have limited knowledge of how different immune cells communicate and coordinate their activities during treatment. This environment, known as the tumor microenvironment, contains many immune cells that can either support anti‐cancer defenses or help tumors evade them. Understanding how these immune cells interact and communicate during treatment could reveal new ways to improve patient outcomes.

In this study, researchers examined tumor samples from patients with stomach cancer, including previously untreated (treatment‐naïve) tumors as well as tumors from patients who had received chemotherapy alone, chemotherapy combined with the immunotherapy drug nivolumab, or chemotherapy combined with targeted therapy. Using single‐cell sequencing, a technique that analyzes individual cells in great detail, the team tracked changes in thousands of immune cells. They found that the combination of chemotherapy and nivolumab produced the most striking effects. A particular group of neutrophils, a type of white blood cell often associated with early immune responses, became more mature and activated after treatment. These neutrophils displayed features associated with antigen presentation and released signals that strongly influenced macrophages, another important immune cell type. As a result, macrophages shifted toward a more inflammatory, M1‐like state that is generally associated with stronger anti‐tumor activity.

Together, these findings show that successful chemo‐immunotherapy may depend not only on activating cancer‐fighting T cells, but also on cooperation between different types of immune cells. Experiments in a mouse tumor model confirmed that removing neutrophils weakened the benefits of combined treatment and reduced the presence of cancer‐fighting macrophages. The study highlights a previously underappreciated partnership in which activated neutrophils help reprogram macrophages toward a cancer‐fighting state, helping to strengthen anti‐tumor immunity. By better understanding and potentially enhancing this interaction, researchers may be able to develop improved treatment strategies, identify patients most likely to benefit from immunotherapy, and discover new targets for future cancer therapies.

https://onlinelibrary.wiley.com/doi/full/10.1111/cas.70384.

## Tumor‐Derived Extracellular Vesicles in Plasma for Predicting Anti‐PD‐1 Antibody Efficacy in Non‐Small Cell Lung Cancer

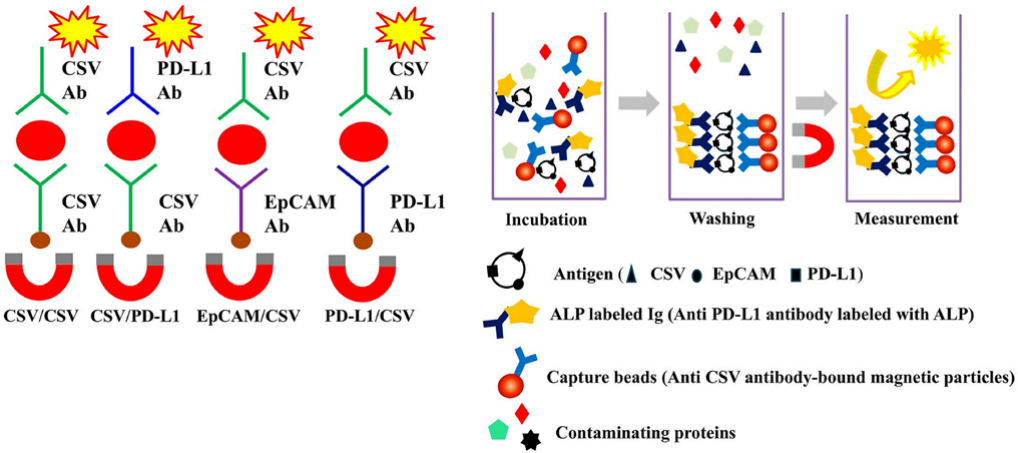

Lung cancer remains one of the world's deadliest cancers, and choosing the right treatment for each patient is still challenging. While tissue samples collected during surgery or biopsy are used to understand the local tumor microenvironment, these procedures can be invasive and cannot always be repeated. This necessitates the development of simple blood tests that provide reliable information about cancer, help detect the disease earlier, and predict how well patients will respond to treatment. Recently, extracellular vesicles (EVs), small particles naturally released by cells into the bloodstream, have been exploited to reveal important clues about lung cancer.

In this context, researchers developed a new laboratory test that measures three different groups of EVs based on proteins found on their surface. These proteins include cell surface vimentin (CSV), epithelial cell adhesion molecule (EpCAM), and programmed cell death ligand 1 (PD‐L1). To evaluate their potential as biomarkers, researchers compared blood samples from patients with non‐small cell lung cancer (NSCLC) with those from healthy volunteers.

Notably, EpCAM/CSV‐EV was particularly effective at identifying patients with early‐stage disease and monitoring disease recurrence, while CSV/CSV‐EV was linked to postoperative outcomes. The team also found that CSV/PD‐L1‐EV could help predict which patients with advanced lung cancer were more likely to benefit from treatments that strengthen the immune system by blocking the PD‐1 pathway.

Together, these findings suggest that the newly developed liquid biopsy, based on the measurement of EV levels in blood samples, could complement existing tests used for lung cancer diagnosis. Such an approach may support earlier diagnosis, help estimate the risk of cancer recurrence after surgery, and improve immunotherapy treatment decisions for patients with NSCLC.

https://onlinelibrary.wiley.com/doi/full/10.1111/cas.70412.

## MAFF Suppresses Necroptosis in Pancreatic Cancer via the GATA4‐MLKL Axis

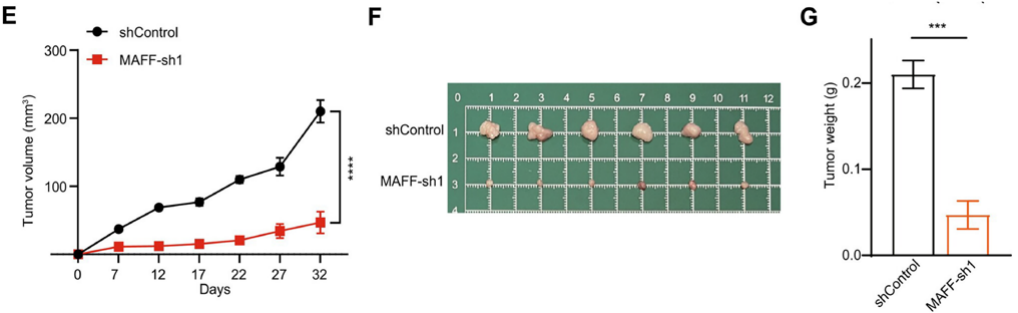

Pancreatic adenocarcinoma (PAAD) is a highly malignant cancer characterized by aggressive proliferation and resistance of apoptosis, contributing to its poor prognosis. Despite advances in cancer therapeutics, the molecular mechanisms underlying these features remain incompletely understood. The musculoaponeurotic fibrosarcoma (MAF) family of transcription factors—regulators of key processes including cell division and apoptosis—has emerged as a potential area of interest. In this study, the researchers identified the small MAF protein, MAFF, as a critical oncogenic driver in PAAD and investigated its role in sustaining tumor proliferation while evading cell death.

The researchers found out MAFF is overexpressed in clinical samples. Artificially reducing MAFF expression decreased the proliferation, survival, and DNA replication, underscoring its role in maintaining PAAD's tumorigenic potential.

Transcriptomic analysis of MAFF‐depleted cells revealed elevated levels of mixed lineage kinase domain like pseudokinase (MLKL), a mediator of necroptosis—a cell death pathway. The promoter region of MLKL comprised a binding site for GATA4, which is a known gene expression activator. Consistently, MAFF depletion increased the expression of GATA4. Conversely, artificial MAFF overexpression produced the opposite effects. Together, these findings indicate that MAFF promotes pancreatic cancer cell survival by inhibiting necroptosis through the suppression of GATA4‐MLKL signaling.

To confirm the cell‐level findings at the organism level, mice were injected with MAFF‐depleted pancreatic cancer cells. These injected cancer cells will develop into tumors over time. MAFF depletion decreased tumor growth, volume, and weight and activated necroptosis‐associated MLKL in mice. Similar to cell experiments, opposite effects were observed when mice were injected with MAFF‐overexpressing pancreatic cancer cells.

To validate the relevance of these findings for human patients, the transcriptome of PAAD from human patients was analyzed. PAAD tissue samples exhibited enhanced MAFF levels. This increase in MAFF expression was correlated with poor survival outcomes.

These findings have real‐world implications. Clinicians can utilize MAFF levels as a proxy to determine the prognosis of patients with PAAD. More importantly, MAAF can be pharmacologically targeted to treat PAAD.

https://onlinelibrary.wiley.com/doi/full/10.1111/cas.70414.

